# Construction of artificial neural network diagnostic model and analysis of immune infiltration for periodontitis

**DOI:** 10.3389/fgene.2022.1041524

**Published:** 2022-11-15

**Authors:** Junwei Xiang, Wenkai Huang, Yaodong He, Yunshan Li, Yuanyin Wang, Ran Chen

**Affiliations:** College and Hospital of Stomatology, Anhui Medical University, Key Lab of Oral Diseases Research of Anhui Province, Hefei, China

**Keywords:** periodontitis, neural networks, machine learning, gene expression, biomarkers

## Abstract

**Background:** Periodontitis is a chronic inflammatory disease leading to tooth loss in severe cases, and early diagnosis is essential for periodontitis prevention. This study aimed to construct a diagnostic model for periodontitis using a random forest algorithm and an artificial neural network (ANN).

**Methods:** Gene expression data of two large cohorts of patients with periodontitis, GSE10334 and GSE16134, were downloaded from the Gene Expression Omnibus database. We screened for differentially expressed genes in the GSE10334 cohort, identified key periodontitis biomarkers using a Random Forest algorithm, and constructed a classification artificial neural network model, using receiver operating characteristic curves to evaluate its diagnostic utility. Furthermore, patients with periodontitis were classified using a consensus clustering algorithm. The immune infiltration landscape was assessed using CIBERSOFT and single-sample Gene Set Enrichment Analysis.

**Results:** A total of 153 differentially expressed genes were identified, of which 42 were downregulated. We utilized 13 key biomarkers to establish a periodontitis diagnostic model. The model had good predictive performance, with an area under the receiver operative characteristic curve (AUC) of 0.945. The independent cohort (GSE16134) was used to further validate the model’s accuracy, showing an area under the receiver operative characteristic curve of 0.900. The proportion of plasma cells was highest in samples from patients with period ontitis, and 13 biomarkers were closely related to immunity. Two molecular subgroups were defined in periodontitis, with one cluster suggesting elevated levels of immune infiltration and immune function.

**Conclusion:** We successfully identified key biomarkers of periodontitis using machine learning and developed a satisfactory diagnostic model. Our model may provide a valuable reference for the prevention and early detection of periodontitis.

## Introduction

Periodontitis, one of the most common oral diseases, is associated with plaque biofilms and accompanied by periodontal attachment loss and alveolar bone resorption (Kinane et al., 2017). Without proper diagnosis and appropriate treatment, persistent inflammation can lead to further tissue destruction, bone resorption, and eventual tooth loss (Tonetti et al., 2018). In addition, periodontitis is a direct manifestation of systemic diseases, independently associated with multiple chronic inflammatory diseases, and may trigger or exacerbate comorbidities (Genco et al., 2020; Hajishengallis et al., 2021). Currently, periodontitis diagnosis is based on a comprehensive examination of the periodontal tissue, including gingival condition, tooth mobility, probing depth, probing bleeding, attachment loss, and bone resorption, supported by radiography (Cafiero et al., 2013). Existing clinical diagnostics for periodontitis can reflect the disease severity and previous periodontal destruction rather than current activity and future progress (Savage et al., 2009). Periodontal treatment strategies may be reactionary and lag behind disease progress because clinicians first respond after the infection is present. Therefore, there is an urgent need to develop better methods for early diagnosis, improve the accuracy of early detection, and better assess the grade of periodontitis.

With the rapid development of microarray screening and high-throughput sequencing, bioinformatics analysis carries great significance in exploring the mechanisms, diagnosis, and prediction of prognosis of periodontitis. Biomarkers are biological indicators with high diagnostic and prognostic value, indicating various stages of periodontitis and providing help in its prevention and treatment (Cafiero et al., 2013). Matrix metalloproteinase-8 (MMP8) and interleukin (IL)-1beta, the most studied biomarkers in the periodontitis field, demonstrate convincing clinical diagnostic validity (Arias-Bujanda et al., 2020). One study identified several biomarkers using bioinformatics analysis, such as CSF3, CXCL12, IL-1B, MS4A1, PECAM1, and TAGLN, and they all served as predictors of diagnosis and prognosis in chronic periodontitis (Suzuki et al., 2019). The combined use of multiple biomarkers can significantly improve the accuracy of classification models compared to using individual markers (Wu et al., 2018; Jin et al., 2020). Jin et al. successfully constructed and validated a 17-miRNA diagnostic signature for periodontitis, showing convincing sensitivity and specificity (Jin et al., 2020).

Research into periodontitis diagnosis based on mRNA expression has certain limitations; most studies use single-marker screens, and classification models with multiple indicators have not yet been constructed (Ji et al., 2022; Song et al., 2015; Suzuki et al., 2019). In addition, selecting key indicators or features is a significant challenge for disease diagnosis model construction. Machine learning algorithms, such as random forest (RF) and artificial neural networks (ANN), can provide new insights into this problem. As one of the essential machine learning algorithms, RF has the advantages of simple operation, high accuracy, and resistance to over-fitting, which helps to identify key features (Wu et al., 2022). ANN, another classical machine learning algorithm, has demonstrated powerful capacities in the processing of medical data (Grobman et al., 2006). The combined use of RF and ANN has been reported efficient in the diagnosis of myocardial infarction, Alzheimer’s disease, and heart failure (Sun et al., 2022; Tian et al., 2020; Wu et al., 2022).

**FIGURE 1:**
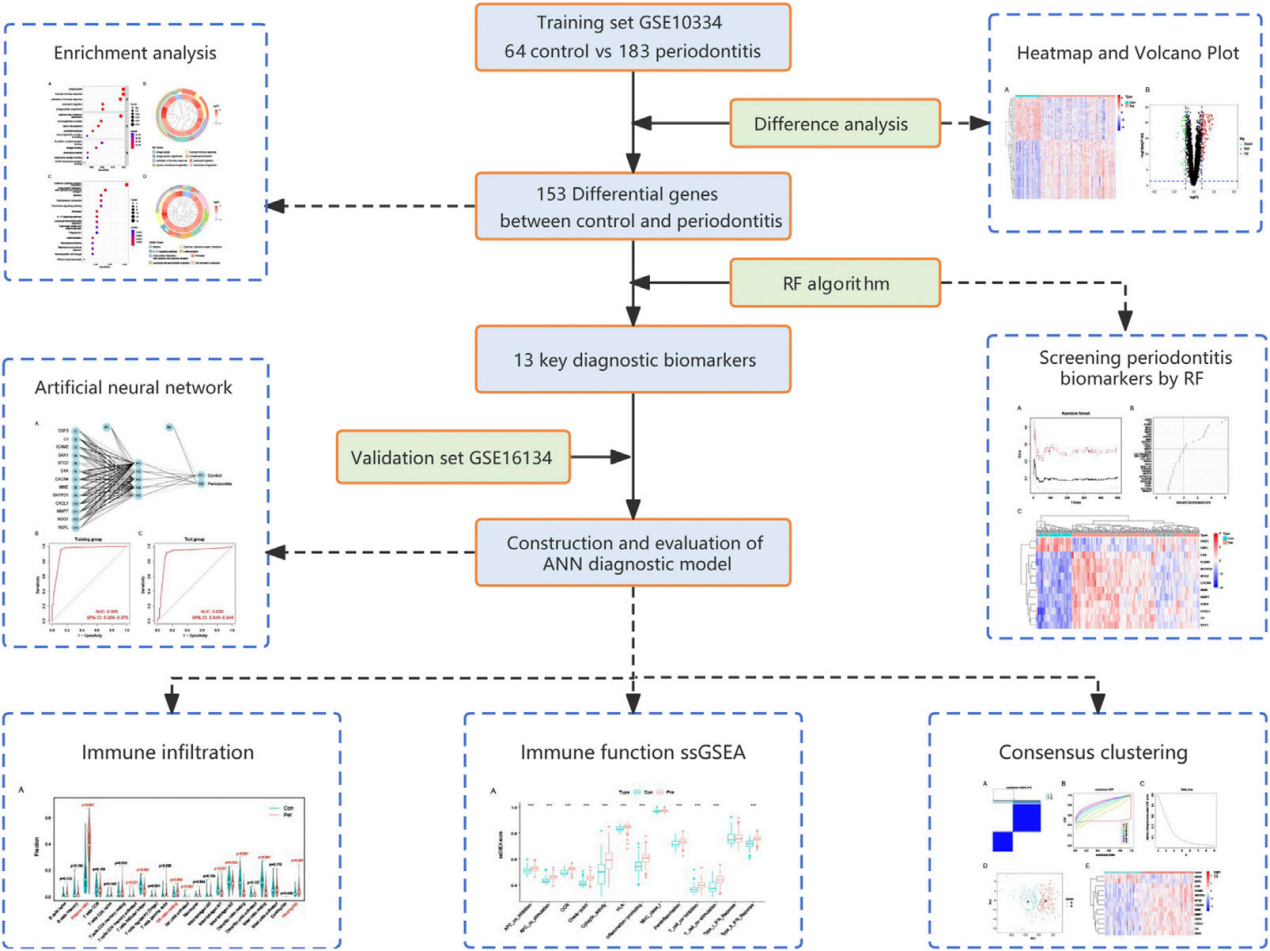
Flow chart of the present study. DEGs, differentially expressed genes; RF, random forest; ANN, artificial neural network.

In this study, we combined RF and ANN to develop a multi-mRNA diagnostic model using Gene Expression Omnibus (GEO) periodontitis expression data (see the analysis process in Figure 1). The diagnostic model had high accuracy and could serve as a tool for the early diagnosis of periodontitis.

## Materials and methods

### Data acquisition

Cases with identified periodontitis samples were included in the experimental group, and healthy samples were included in the control group. Periodontitis was defined according to the case definition proposed at the 2017 World Workshop on the Classification of Periodontal and Peri-Implant Diseases: 1) interdental clinical attachment loss detectable at ≥ 2 nonadjacent teeth or 2) buccal or oral clinical attachment loss with pocketing >3 mm detectable at ≥ 2 teeth (Tonetti et al., 2018). First, we searched and downloaded two RNA expression datasets from the GEO database using the keyword “periodontitis”. GSE10334 contained 64 healthy and 183 periodontitis samples, and GSE16134 contained 69 healthy and 241 periodontitis samples, all of which were processed using the GPL570 platform of the Affymetrix Human Genome U133 Plus 2.0 Array. Based on available literature on using machine learning in disease diagnosis, we believed that the sample sizes of these two datasets were appropriate. The obtained RNA-Seq data were then annotated and normalized using R software (v4.1.2). We selected GSE10334 as the training cohort and GSE16134 as the validation cohort.

### Differential expression and functional enrichment analysis

The differentially expressed genes (DEGs) between the periodontitis and control groups in the training set were identified using the “limma” R package, with |logFC| > 1.0 and *p*-values < 0.05 as the screening criteria (Ritchie et al., 2015). DEGs were visualized using the “pheatmap” and “ggplot2” R packages. We then performed Gene Ontology (GO) and Kyoto Encyclopedia of Genes and Genomes (KEGG) enrichment analyses on the DEGs using the “clusterProfiler” R package (Yu et al., 2012). The top enriched functions or pathways were then displayed in bubble and circle plots.

### Key biomarkers screening with random forest

We employed the “randomForest” R package for further DEG screening (Lawrence et al., 2006). First, the error rates using 1–500 trees were calculated. We comprehensively evaluated the error rates and stability to select the optimal tree number, usually that with the lowest error rate and the best stability. Next, an RF model was constructed with the optimal tree number, and potential periodontitis biomarkers were identified based on the mean decrease in Gini coefficient. We defined genes with importance greater than 2 as key biomarkers, which is a common screening criterion in RF algorithms and has been used in similar studies (Tian et al., 2020; Wu et al., 2022). Finally, we performed unsupervised hierarchical clustering on the above biomarkers.

### Construction and evaluation of artificial neural networks diagnostic model

For the construction of the ANN diagnostic model, we first used the min–max method to normalize the input data, converting the expression data of 13 key biomarkers into gene scores. The biomarker expression level for each sample was compared to the median value of all samples. If the expression level of an upregulated gene was greater than the median value, its gene score was defined as one; otherwise, it was 0. Similarly, if the expression level of a downregulated gene was less than the median value, it was defined as 1. We then utilized the “neuralnet” R package to calculate the gene weight and establish the ANN classification model (Guenther et al., 2010). The ANN model consisted of one input layer, one hidden layer, and one output layer. To further evaluate the model performance, we calculated the area under (AUC) the receiver operating characteristic (ROC) curve of the training set using the “pROC” R package (Robin et al., 2011). The model was also validated using another patient cohort, GSE16134.

### Immune infiltration analysis

CIBERSORT is a deconvolution algorithm quantifying cell types based on gene expression profiles and can assess the distribution of 22 immune cells in tissues (Newman et al., 2015). We used CIBERSORT to comprehensively analyze the immune infiltration landscape in the GSE10334 cohort, using waterfall and violin plots to show the differences between the control and periodontitis groups. Furthermore, we calculated enrichment scores for immune cells and functions using single-sample Gene Set Enrichment Analysis (ssGSEA; Haenzelmann et al., 2013). Heatmaps were used to show the association of key periodontitis biomarkers with immune cells and immune functions, respectively.

### Unsupervised clustering of periodontitis patients

Based on the expression of key biomarkers, we applied the “ConsensusClusterPlus” R package to perform unsupervised cluster analysis on the training cohort to identify potential molecular subtypes. The k-means algorithm with 1,000 iterations and an 80% resampling rate was used to guarantee classification stability. The t-distributed stochastic neighbor embedding (tSNE) analysis was utilized to verify the classification accuracy and visualized using the “ggplot2″ R package.

### Statistical analysis

The Wilcoxon signed-rank test was used to analyze differences in immune scores between the control and periodontitis groups. The differences between two molecular subgroups were also analyzed using the Wilcoxon signed-rank test. Spearman’s rank correlation coefficients were used to explore the correlation between periodontitis biomarker expression and immune cells or immune functions. Statistical analyses were performed using R. Unless otherwise stated, *p* < 0.05 was considered statistically significant.

## Results

### Identification of differentially expressed genes in periodontitis

**FIGURE 2:**
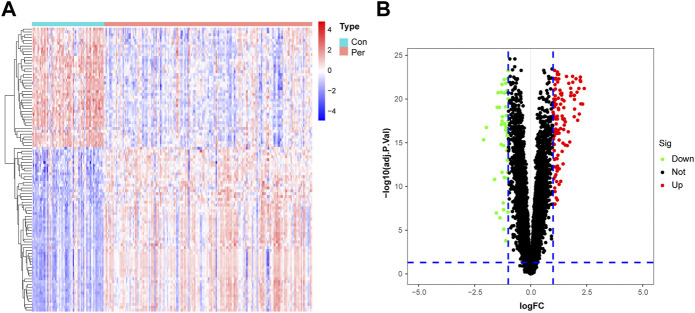
Identification of DEGs in the training cohort. **(A)** The heatmap of the 153 DEGs, including 111 up-regulated and 42 down-regulated ones. **(B)** Volcano plots of all DEGs in the GSE10334 dataset. Con, control group; Per, periodontitis group.

A flow chart of the study process is presented in Figure 1. All analysis code is provided in Supplementary Table S1. After processing the data, a total of 153 DEGs were identified using the “limma” R package, including 111 significantly upregulated genes and 42 significantly downregulated genes. As shown in Figure 2A, these genes were significantly different between the control and periodontitis groups. Volcano plots further suggested the expression status and statistical significance of all DEGs (Figure 2B).

### Functional enrichment of differentially expressed genes

**FIGURE 3:**
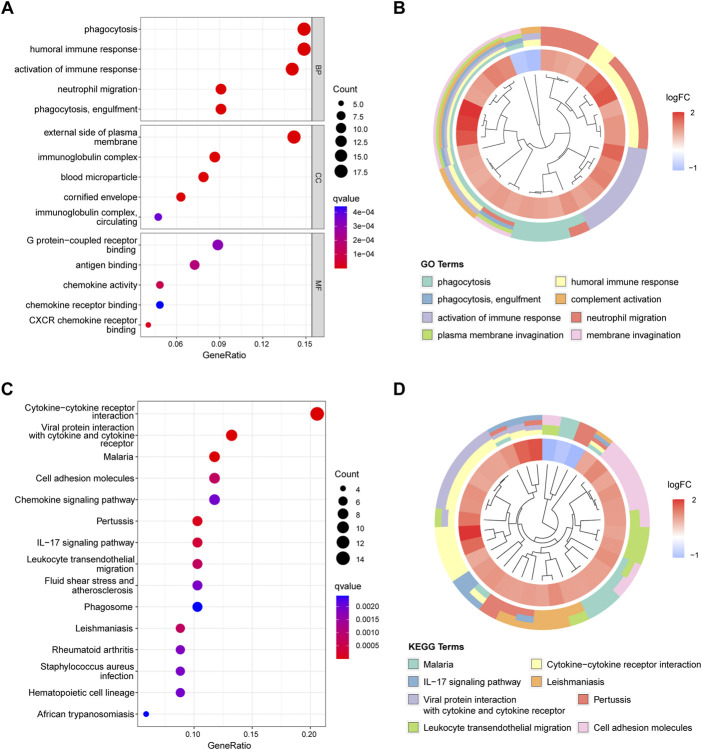
Figure 3, Functional enrichment analysis results. **(A)** Top five enriched GO terms in biological process (BP), cell components (CC), and molecular function (MF). **(B)** Top eight enriched GO terms difference in periodontitis. **(C)** Top 15 enriched KEGG signaling pathways. **(D)** Top eight enriched KEGG pathways difference in periodontitis.

We used GO and KEGG enrichment analyses to determine the functions of these genes. GO analysis revealed that the DEGs mainly regulated immune-related functions such as phagocytosis, humoral immune response, activation of the immune response, and neutrophil migration (Figures 3A,B). Meanwhile, KEGG analysis suggested that these genes were significantly enriched in immune pathways such as cytokine−cytokine receptor interaction, viral protein interactions with cytokines and cytokine receptors, cell adhesion molecules, chemokine signaling pathways, and the IL-17 signaling pathway (Figures 3C,D).

### Screening for diagnostic biomarkers

**FIGURE 4:**
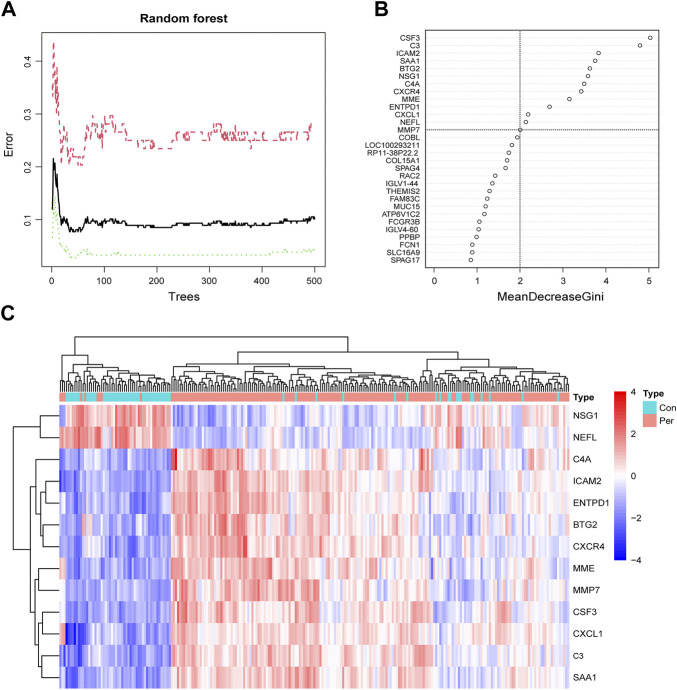
Screening periodontitis biomarkers by random forest. **(A)** The correlation plot between the RF trees number and error rate. The *x*-axis is the number of trees, and the *y*-axis is the error rate of cross-validation. The red curve represents the treat group, the green curve represents the control group, and the black curve represents all samples. **(B)** The Gini coefficient method in random forest modeling of the training cohort. The importance index is on the *x*-axis, and the genetic variable is on the *y*-axis. **(C)** Heatmap of the 13 key periodontitis biomarkers.

To screen for reliable diagnostic biomarkers of periodontitis, we entered the DEG gene scores into the RF model. According to the relationship between the RF tree number and the model error rate, we chose the tree number corresponding to the lowest error rate (n = 34; Figure 4A). Figure 4B shows the top 30 genes in the RF classifier; CSF3 was the most important biomarker. Finally, we obtained a total of 13 key periodontitis biomarkers using importance >2 as the screening criterion. The heatmap revealed that NSG1 and NEFL were downregulated genes in the periodontitis group, and the remaining 11 were upregulated (Figure 4C). Each marker exhibited excellent diagnostic performance, with the lowest AUC of 0.831 and the highest of 0.916 (Supplementary Figure S1).

### Artificial neural networks diagnostic model construction and validation

**FIGURE 5:**
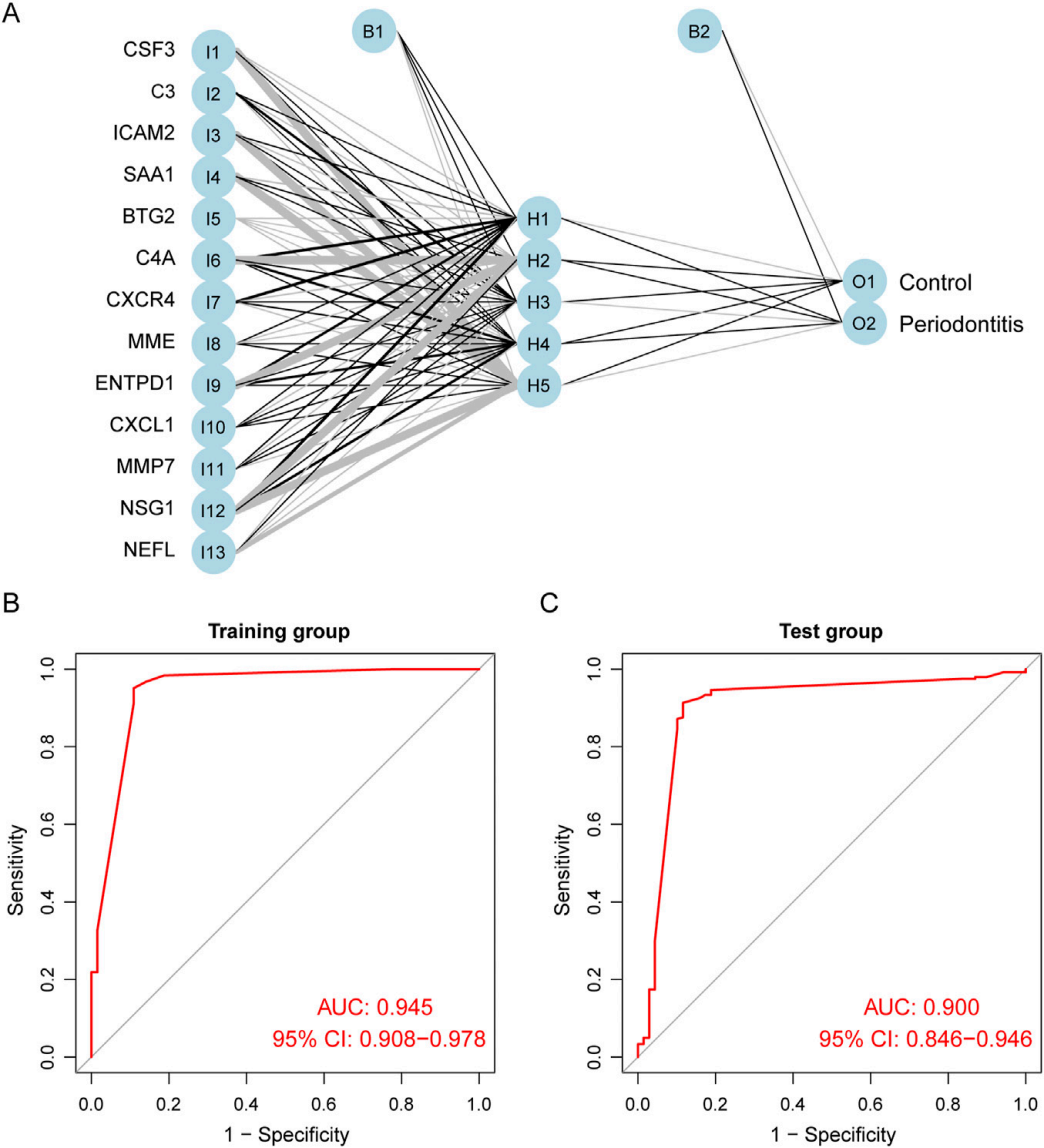
Construction and evaluation of ANN diagnostic model. **(A)** The visualization of the artificial neural network. **(B)** ROC curves of the training group. **(C)** ROC curves of the test group.

We used ANN to analyze the weights of the 13 biomarkers based on gene scores. The ANN diagnostic model consisted of 13 input, five hidden, and two output parameters (Figure 5A). The weights of each biomarker are shown in Supplementary Table S2. The entire training was performed for 2,304 steps, and the absolute partial derivative of the error function was less than 0.01. We then evaluated the model performance using the “pROC” R package, with an AUC of 0.945 in the training cohort, indicating that the model had excellent classification accuracy (Figure 5B). In addition, the ANN model also demonstrated superior performance in the validation cohort, GSE16134; the AUC was 0.900 (Figure 5C). As shown in Supplementary Table S3, we adjusted the importance threshold of RF to determine the optimal model. The results suggested that our model performed optimally, possessing the lowest AUC reduction with AUCs greater than 0.90.

### Immune infiltration assessment

**FIGURE 6:**
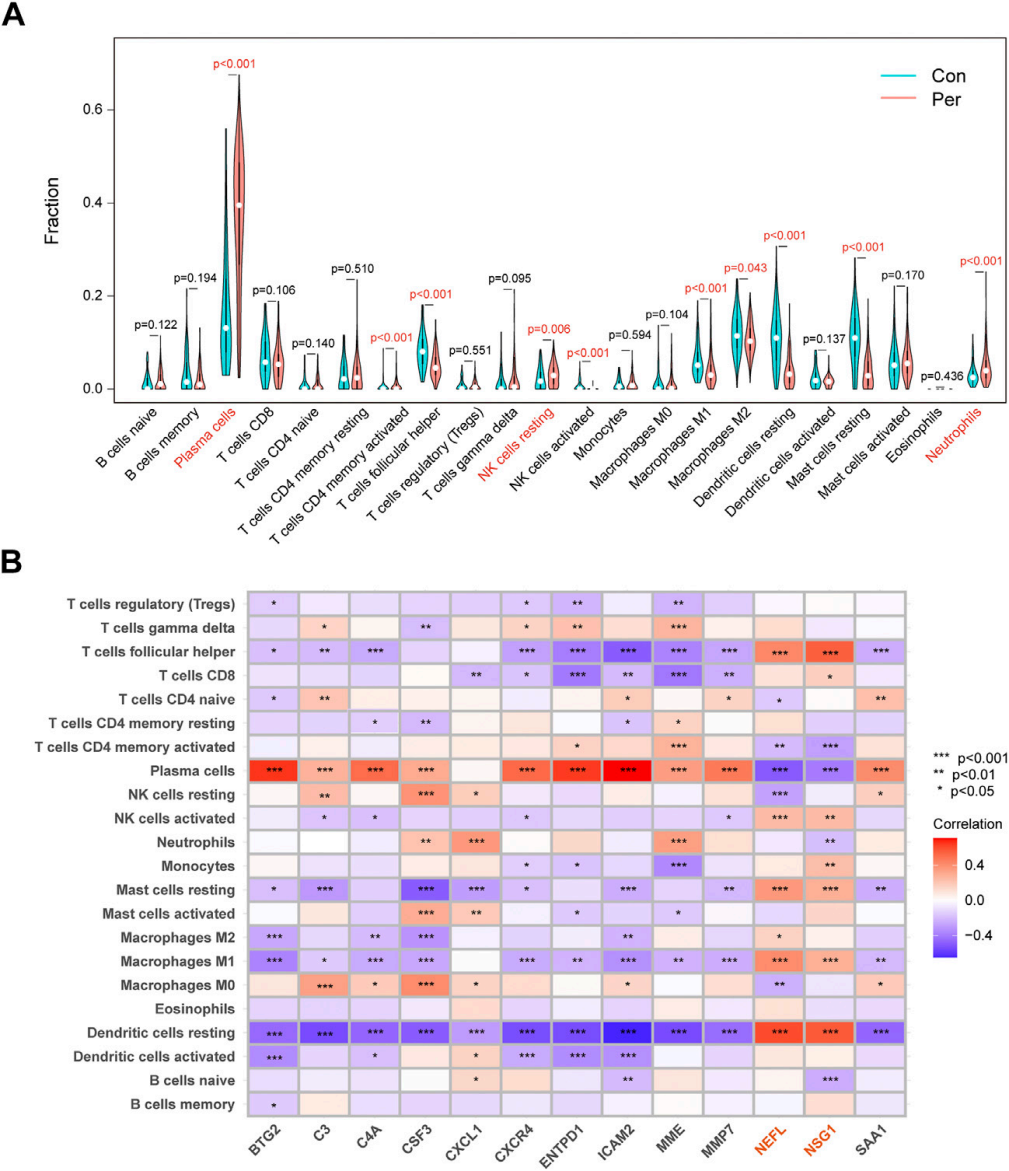
Immune infiltration differences and correlations. **(A)** Violin plots of the 22 immunocytes differences between control and periodontitis groups. **(B)** Correlation matrix of 13 key biomarkers and immunocytes distribution. *p < 0.05, **p < 0.01, ***p < 0.001.

We used CIBERSORT to assess the distribution of 22 immune cells, and plasma cells were the main cell type in periodontitis samples (Supplementary Figure S2). The relative immune cell scores for each sample are displayed in Supplementary Table S4. As shown in Figure 6A, the proportions of plasma cells, resting natural killer cells, and neutrophils in the periodontitis group were significantly higher than in the control group. Looking at the heatmap of the correlation between biomarker expression and immune cell scores, we found that NEFL and NSG1 differed from other biomarkers (Figure 6B). The expression of NEFL and NSG1 was significantly positively correlated with T follicular helper cells, resting mast cells, M1 macrophages, and resting dendritic cells, while it was significantly negatively correlated with plasma cells. Notably, these biomarkers were all associated with immune infiltration in periodontitis.

### Immune function analysis

**FIGURE 7:**
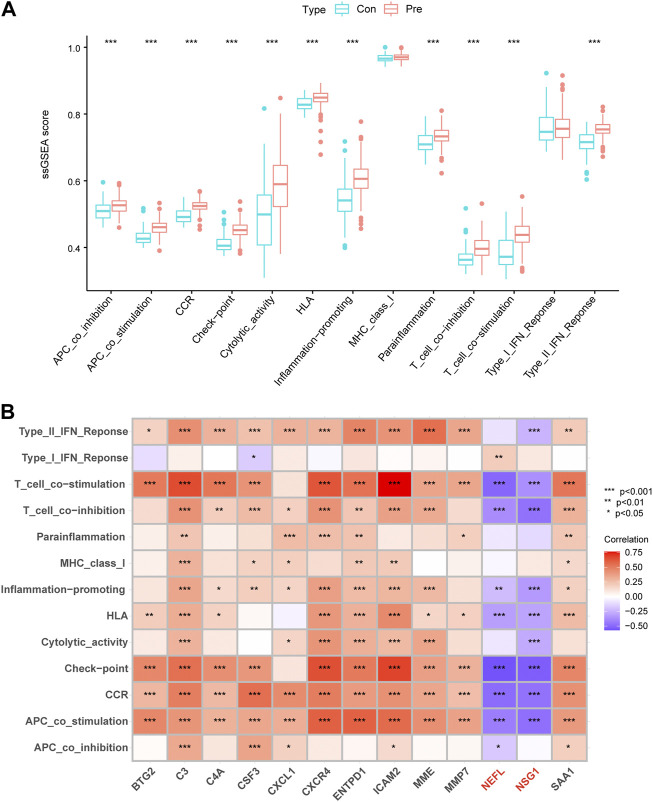
Immune functions differences and correlations. **(A)** Box plots of the 14 immune function differences between control and periodontitis groups. **(B)** Correlation matrix of 13 key biomarkers and immune functions. *p < 0.05, **p < 0.01, ***p < 0.001.

We employed ssGSEA to calculate immune function enrichment scores (Supplementary Table S5). The enrichment score of the periodontitis group was significantly higher than the control group, indicating that periodontitis had more active immune processes (Figure 7A). NEFL and NSG1 were negatively correlated with the 14 immune function scores, while other biomarkers were positively correlated (Figure 7B). The results suggested that NEFL and NSG1 may be negative immune regulators in periodontitis, while other biomarkers may be active immune regulators.

### Identification of immune characteristics in periodontitis subgroups

**FIGURE 8:**
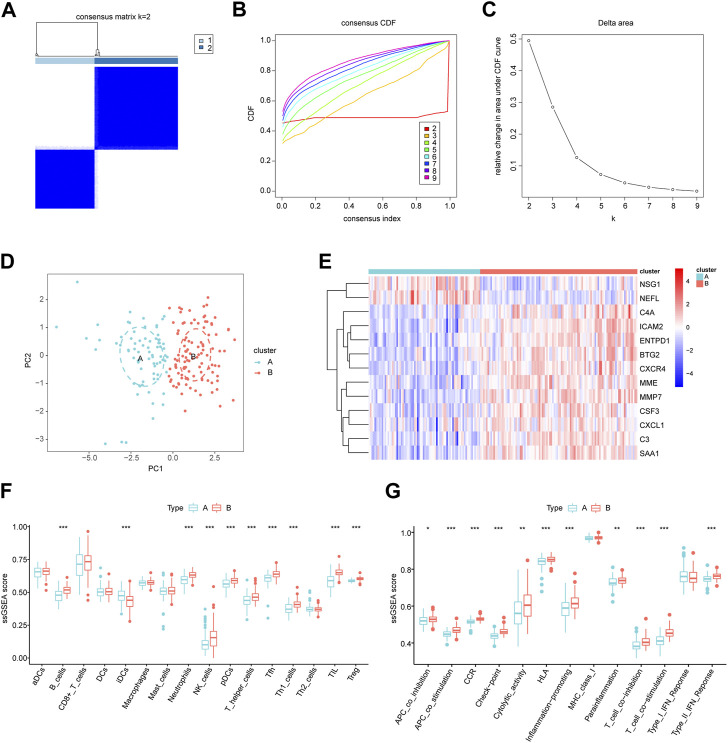
Identification of molecular subgroups in periodontitis. **(A)** Consensus clustering matrix when k = 2. **(B)** The cumulative distribution function (CDF) from k = 2 to 9. **(C)** Relative variation of the area under the CDF region at k = 2–9. **(D)** The t-SNE diagram for verifying the differences between two modification subgroups. **(E)** Heatmap of 13 key biomarkers between two subgroups. **(F,G)** The differences in infiltrated immune cells and functions. *p < 0.05, **p < 0.01, ***p < 0.001.

Patients with periodontitis were classified using consensus clustering to gain better insight into the roles of biomarkers in disease development. The results suggested that, when the number of clusters (k) was 2, the periodontitis samples in the consensus matrix obtained the best clustering, with the highest intra-omic correlation and the least inter-omic interference (Figures 8A–C). Therefore, we divided the periodontitis group into two subgroups, defined as cluster A (n = 76) and cluster B (n = 107). tSNE analysis further demonstrated a significant distribution difference between the two subgroups (Figure 8D). A heatmap revealed differences in biomarker expression between the two subgroups, with active immune genes highly expressed in cluster B (Figure 8E). Interestingly, NSG1 and NEFL were again distributed differently from other markers and more likely to be drivers of consensus clustering. Furthermore, we observed alternations in immune infiltration between cluster A and B, and cluster B had a higher distribution of immune cells (Figure 8F). Most immune activation, inflammatory responses, human leukocyte antigens, and immune checkpoints were also significantly enhanced in cluster B (Figure 8G). These results suggested that cluster B had high immune system activity, which may potentially benefit from immunosuppressive therapy.

## Discussion

Periodontitis is not only a common cause of severe tooth loss but also a driver and direct manifestation of several diseases, such as diabetes, cancer, cardiovascular disease, and rheumatoid disease (Genco et al., 2020). Early diagnosis of periodontitis can preserve teeth and chewing ability and significantly improve patient outcomes. Periodontitis is determined by the clinical status of the periodontal tissue and supplemented by imaging features (Tonetti et al., 2018). However, the early detection of periodontitis is unsatisfactory due to the inevitable error in periodontal exploration using measurement of clinical attachment. When using periodontal probing for diagnosis, sustained clinical attachment loss must occur before a site can be considered periodontitis. This approach is an assessment of accumulated past disease and requires following the patient for several years, which may miss the optimal timing for early diagnosis and treatment (Korte et al., 2016). In addition, imaging examinations based on alveolar bone loss are not specific enough, and mild and moderate periodontitis is missed (Tonetti et al., 2018). Therefore, objective and quantitative methods for the early diagnosis of periodontitis are urgently needed. Advances in machine learning have enabled the development of using biomarkers for disease diagnosis and prognosis (Duan et al., 2022; Wu et al., 2022).

Papantonopoulos et al. first applied an ANN algorithm to develop a classification model for chronic and aggressive periodontitis based on immune parameters (Papantonopoulos et al., 2014). Shimpi et al. analyzed five machine learning methods, including RF and ANN, and developed a clinical feature-based periodontitis risk assessment model (Shimpi et al., 2020). The above two models can assist clinical treatment decisions, but they are not useful for early diagnosis. Furthermore, these studies focused on key phenotypic characteristics, whereas the current study further explored the diagnosis of periodontitis at the molecular level. We also established larger discovery and validation cohorts to ensure generalizability and accuracy of the biomarkers. Previous literature suggested that combing multiple biomarkers can improve model accuracy (Jin et al., 2020); therefore, we constructed a model for periodontitis diagnosis with multiple mRNA markers using two machine learning algorithms, RF and ANN.

The present study first identified 153 DEGs between periodontitis and healthy samples from the GEO dataset. Gene enrichment analysis showed that these genes were mainly involved in phagocytosis, humoral immune response, immune response activation, neutrophil migration, cytokine interaction, cell adhesion molecules, and the IL-17 signaling pathway. These results suggested that the DEGs are actively involved in inflammatory processes in periodontitis and may be critical for its development. The RF classifier screened 34 potential markers and obtained 13 key periodontitis biomarkers. We found that these key markers were associated with periodontitis, immune cells, or apoptosis. CSF3, ICAM2, and MMP7 serve as diagnostic and prognostic biomarkers for periodontitis (Ji et al., 2022; Lundmark et al., 2017; Suzuki et al., 2019). C3, C4A, and ENTPD1 are closely associated with periodontitis severity and may regulate periodontitis occurrence and development. C3 mediates Porphyromonas gingivalis-induced periodontal inflammation and bone loss (Maekawa et al., 2014). C4A encodes the classical complement factor C4, and patients with C4 deficiency are more prone to severe chronic periodontitis (Seppanen et al., 2007). High expression of ENTPD1 (CD39) relieves the growth inhibition of periodontal ligament cells by ATP (Kawase et al., 2007). Furthermore, MME was upregulated, and the neuron-related gene NEFL was downregulated in periodontitis (Andriankaja et al., 2012; Kim et al., 2016). Periodontal pathogens have been reported to cause neuroinflammation and neurodegeneration in mice (Ilievski et al., 2018). In addition, periodontitis was shown to significantly alter neural consequences when comorbid with diabetes (Flores-Tochihuitl et al., 2021). Therefore, we believe that the decreased expression of NEFL may be due to neural consequences caused by periodontal pathogens.

CRCR4, CXCL1, and SAA1 regulate immune cell distribution and induce inflammatory cell recruitment in periodontal diseases (Hirai et al., 2019; Kim et al., 2022; Korbecki et al., 2022). The machine learning algorithms here detected several recognized periodontitis biomarkers or inflammatory factors, including C3, C4A, CRCR4, and CXCL1, confirming the algorithms’ accuracy. Among the 13 periodontitis biomarkers, BTG2 and NSG1 were identified for the first time. BTG2 and NSG1 play an important role in apoptosis, and apoptosis is clearly involved in periodontitis and can serve as a biomarker (Song et al., 2017). BTG2 regulates the cell cycle and apoptosis and is involved in B cell and thymocyte progenitor differentiation (Yuniati et al., 2019). NSG1 (NEEP21), a p53 transcriptional target, plays an essential role in DNA damage and apoptosis (Ohnishi et al., 2010). Finally, we established an ANN diagnostic model for periodontitis based on 13 key biomarkers. The model could reliably discriminate between periodontitis and healthy samples (AUC ≥0.900) in exclusive training and validation cohorts.

A growing body of studies has shown that different immune cell infiltration is seen in the periodontitis inflammatory microenvironment, which is crucial for its occurrence, development, and treatment (Yang et al., 2021). We found that the immune functions were upregulated in periodontitis, suggesting that immunosuppressant administration is a potential therapeutic strategy. The present study also showed that plasma cells were the most predominant immune cells in periodontal tissue and may play a dominant role in immune regulation. Previous research has also confirmed this. Plasma cells account for about 50% of the total leukocytes in periodontitis lesions and mediate humoral immunity against periodontal pathogens (Thorbert-Mros et al., 2015). When the balance between microbiome invasion, host defense, and tissue regeneration is upset, B cells and plasma cells induce pathological bone resorption, resulting in insufficient bone tissue and ultimately tooth loss (Zouali, 2017). A clinical trial has also shown that anti-B cell depletion therapy significantly reduced clinical periodontal inflammation and tissue destruction in patients with rheumatoid arthritis (Harada et al., 2006). Interestingly, we found that 11 key biomarkers were positively correlated with plasma cell and immune function enrichment scores, suggesting that the effects of these genes in periodontitis may be consistent with the inflammation-promoting role of plasma cells. The expression of these 11 biomarkers was also negatively correlated with T follicular helper cells, M1 macrophages, and resting dendritic cells, and these cells play complex roles in immune regulation in periodontitis (Ebersole et al., 2021; Parisi et al., 2018; Song et al., 2018). The immune-related results of NEFL and NSG1 differed from those of the above 11 biomarkers, indicating that they may play an inhibitory role or be negatively regulated in inflammatory processes. However, more evidence is needed to identify the potential functional link between biomarkers and immune cells, and the relevance of this association is currently unclear. Furthermore, the inflammatory response in periodontitis may alter immune cell distribution, and the expression of biomarkers may be associated with this response. Thus, the causal relationship between immune cell infiltration and biomarker expression should be considered with care. Finally, we performed unsupervised clustering to assess molecular patterns in periodontitis, identifying two distinct molecular subgroups. Immune infiltration analysis showed that cluster B exhibited higher immune infiltration and stronger immune function. Therefore, cluster B may have higher response rates to immunosuppressant therapy and, ultimately, a better prognosis.

In the present study, we applied an innovative combination of RF and ANN algorithms to the early diagnosis of periodontitis, demonstrating excellent diagnostic performance in a large cohort. Although DNA methylation biomarkers and miRNA-based models for periodontitis classification have been developed, these studies used small sample sizes and showed modest performance (Jin et al., 2020; Wang et al., 2021). In addition, we analyzed the association of key biomarkers with inflammatory processes and explained the rationality of marker selection. We believe that using biometric features and machine learning is ideal for the early diagnosis and long-term monitoring of periodontitis. Point-of-care testing platforms based on saliva, gingival crevicular fluid, subgingival samples, and subgingival plaque have shown great potential in the diagnosis of periodontitis. They are simple to operate, fast, and carry a low-cost, allowing for easy screening for periodontitis. The combination of machine learning and big-data analysis can provide more accurate diagnoses and more effective treatments (He et al., 2018). The development of microfluidic technology also has broad application prospects in diagnosing periodontal diseases and predicting periodontal treatment outcomes (Cafiero et al., 2013).

The present study also had several limitations. First, the model input data was provided by gingival tissue samples, and tissue acquisition presents a challenge in clinical practice. Point-of-care testing platforms and microfluidic technology may help identify mRNA signatures in biopsy samples. Second, mechanistic explanations of the correlations between some biomarkers and periodontitis are lacking, and further experimental studies are necessary. Third, a lack of clinical information prevented further exploration of clusters A and B. Furthermore, cellular-level characterization is required to determine whether plasma cells were the predominant cell population in our findings. Finally, more independent patient cohorts should be used to evaluate the ANN classification model’s performance. Our next step is to collect patient tissue samples from an affiliated hospital for verification.

## Conclusion

In summary, we constructed a new periodontitis classification model using on machine learning algorithms, demonstrating satisfactory performance in an independent cohort. In addition, we comprehensively assessed the association of key biomarkers with immune infiltration. We believe that the diagnostic model and biomarkers discussed here may shed new light on the exploration of mechanisms and clinical diagnosis of periodontitis. However, it is noteworthy that further experimental studies and independent patient cohorts are warranted to validate the present results. (Cai and Jiang, 2020), (Feres et al., 2018), (Nagashima et al., 2017) and (Ravida et al., 2020)

## Data Availability

The original contributions presented in the study are included in the article/Supplementary Material, further inquiries can be directed to the corresponding authors.
