# Supplementary material for: Construction of artificial neural network diagnostic model and analysis of immune infiltration for periodontitis
Source: Front Genet. 2022 Nov 15;13:1041524. doi: 10.3389/fgene.2022.1041524 (PMC9705329; doi:10.3389/fgene.2022.1041524)
Supplement: Supplementary file 5 [file DataSheet2.docx]

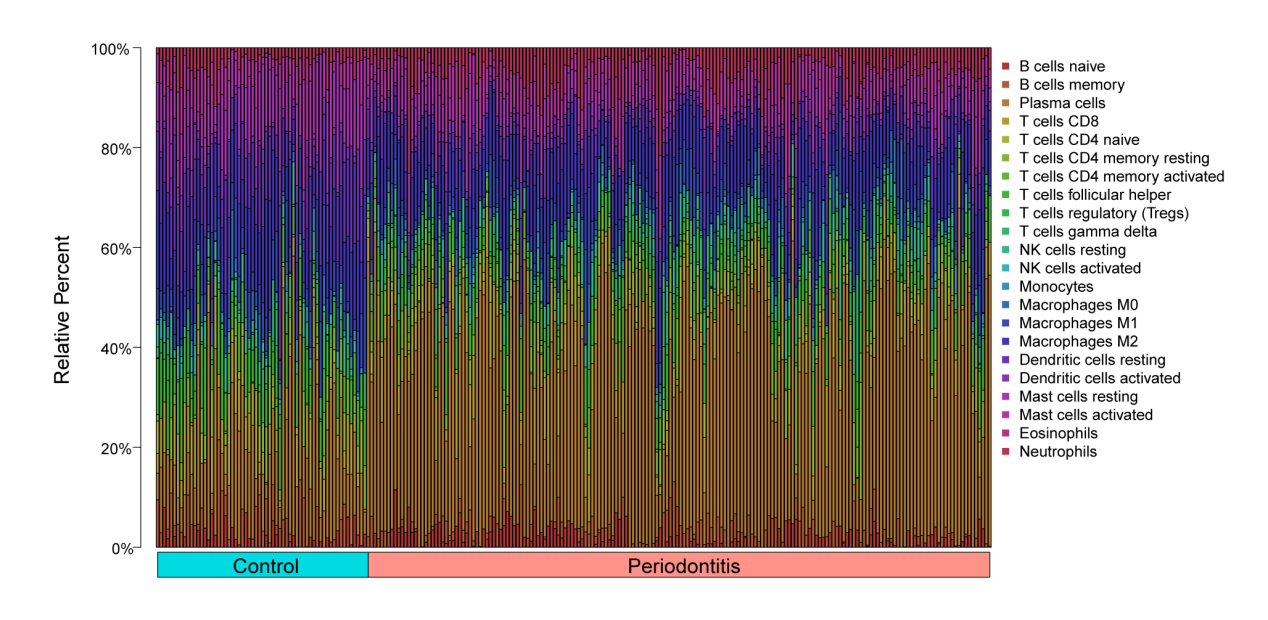


**Supplementary Figure 2.** Bar plots of 22 immunocytes relative distributions in control and periodontitis samples.
