# Supplementary figures and images for: Construction of artificial neural network diagnostic model and analysis of immune infiltration for periodontitis
Source: Front Genet. 2022 Nov 15;13:1041524. doi: 10.3389/fgene.2022.1041524 (PMC9705329; doi:10.3389/fgene.2022.1041524)

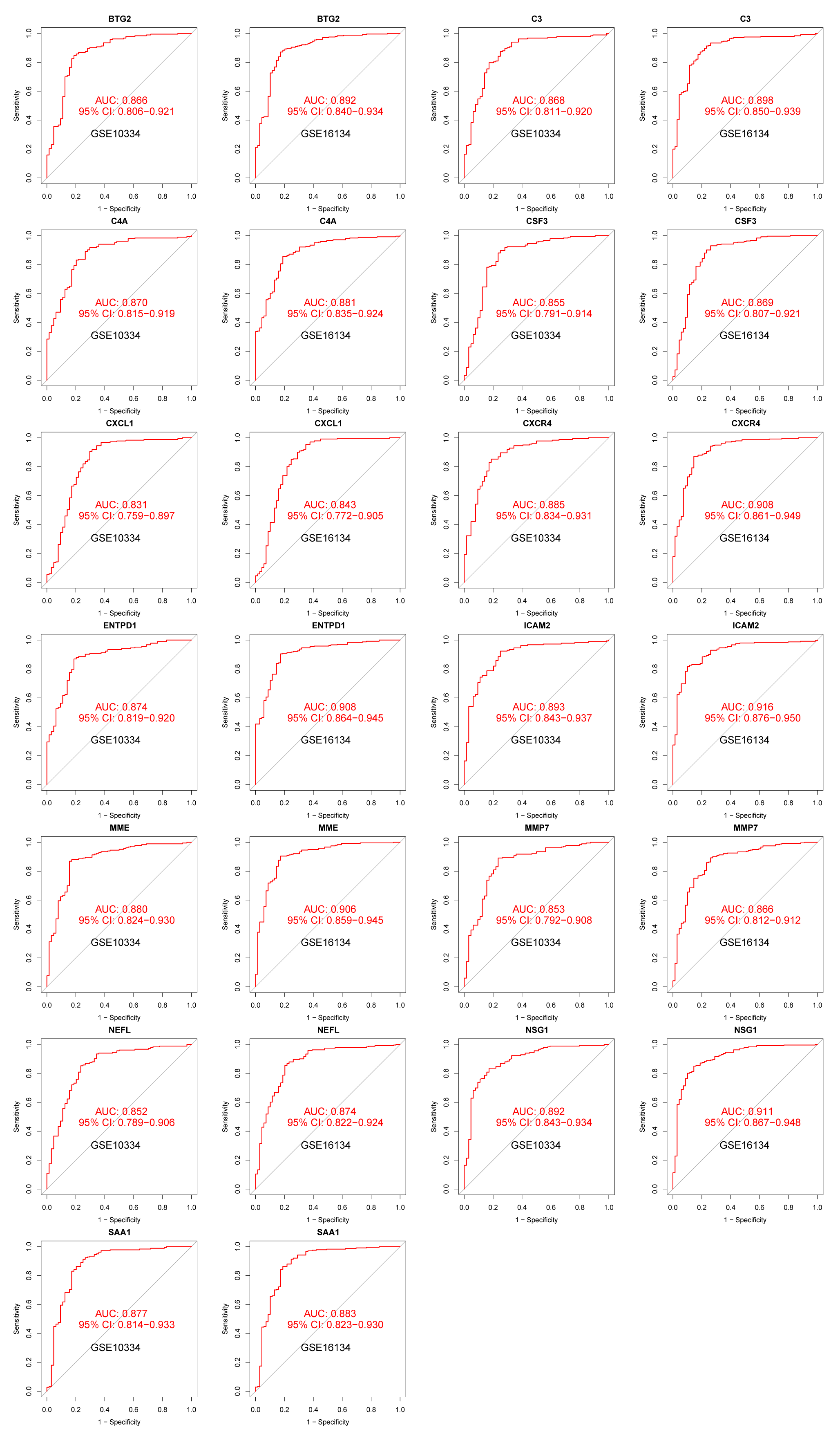


**Supplementary Figure 1.** The diagnostic performance of each marker in two cohorts.

Supplement: Supplementary file 7 [file DataSheet1.docx]
